# Subclinical Cardiac Involvement in Asymptomatic ATTR Mutation Carriers: Insights from Cardiac MRI, Myocardial Strain, and Mapping Techniques

**DOI:** 10.3390/jcdd12050172

**Published:** 2025-05-01

**Authors:** Luca Conia, Daria Filatova, Giacomo Pambianchi, Livia Marchitelli, Giulia Cundari, Giuseppe Stancanelli, Maria Alfarano, Giulia Marchionni, Cristina Chimenti, Carlo Catalano, Nicola Galea

**Affiliations:** 1 Department of Radiological, Oncological and Pathological Sciences, Sapienza University of Rome, 00161 Rome, Italy; luca.conia@uniroma1.it (L.C.); giacomo.pambianchi@uniroma1.it (G.P.); livia.marchitelli@uniroma1.it (L.M.); giulia.cundari@uniroma1.it (G.C.); giuseppe.stancanelli@uniroma1.it (G.S.); carlo.catalano@uniroma1.it (C.C.); 2 Department of Radiation Diagnostics and Therapy, Lomonosov Moscow State University, Moscow 119991, Russia; dariafilatova.msu@mail.ru; 3 Department of Clinical, Internal, Anesthesiologist and Cardiovascular Sciences, Sapienza University of Rome, 00161 Rome, Italy; maria.alfarano@ymail.com (M.A.); cristina.chimenti@uniroma1.it (C.C.)

**Keywords:** amyloidosis, cardiac magnetic resonance, transthyretin, T1 mapping, ECV, myocardial strain

## Abstract

Transthyretin cardiac amyloidosis (ATTR-CA) leads to myocardial infiltration, affecting prognosis and survival. Diagnosing early-stage ATTR-CA remains challenging due to its subtle manifestations. This study investigates subclinical myocardial alterations in asymptomatic ATTR mutation carriers (ATTR-MC) using advanced cardiac magnetic resonance (CMR) techniques, including T1 mapping and myocardial strain analysis. A retrospective cohort of 60 subjects was analyzed, comprising 20 ATTR-CA patients, 20 asymptomatic ATTR-MC, and 20 controls. Standard CMR parameters were compared alongside myocardial strain analysis. Results indicated that despite preserved ejection fraction and myocardial morphology, ATTR-MC exhibited significantly impaired left ventricular global longitudinal strain (LV GLS), left atrial reservoir, conduit, and booster pump strain (LA RS, CS, and BPS) compared to controls. However, native T1 and extracellular volume (ECV) values remained within normal ranges, distinguishing early dysfunction from overt amyloid deposition seen in ATTR-CA. These findings suggest that myocardial strain analysis could serve as an early biomarker for subclinical ATTR-CA, offering a potential target for selecting patients who may benefit from early intervention. Implementing CMR-derived strain parameters in clinical practice may improve risk stratification and timely therapeutic decisions in ATTR-MC.

## 1. Introduction

Systemic amyloidosis comprises a group of infiltrative disorders characterized by the accumulation of misfolded precursor proteins that form cross-β-sheet-rich insoluble amyloid fibrils in the extracellular space of various tissues [1]. Transthyretin (TTR), a protein responsible for transporting thyroxine and retinol-binding protein, can misfold and deposit into various organs and peripheral nerves, which can cause them to function abnormally.

Cardiac amyloidosis (CA), hereditary (h-ATTR-CA) or wild-type (wt-ATTR-CA), is the primary determinant of prognosis in patients with ATTR amyloidosis, with survival rates averaging 4–5 years after diagnosis [2].

Cardiac magnetic resonance (CMR) plays a pivotal role in diagnosing and staging CA, as it provides a non-invasive evaluation of cardiac morphology, function, and extracellular volume expansion, implementing prognostic stratification and therapeutic response evaluation.

Particularly, CMR proves to be crucial in the advanced stages of CA, reliably identifying hallmark features such as ventricular hypertrophy, diffuse late gadolinium enhancement (LGE), elevated native T1 (nT1), and increased extracellular volume (ECV) values [3].

Conversely, diagnosing early-stage CA is particularly challenging due to its subtle manifestations, which may include minimal hypertrophy without distinct morphological or signal alterations, overlapping with other conditions such as hypertensive heart disease. Early diagnosis of CA is crucial for the timely initiation of appropriate therapy, as recent advances in CA treatment have significantly improved survival and quality of life by slowing disease progression and preserving cardiac function. Delayed diagnosis of CA can result in irreversible damage, limiting treatment options and leading to poorer outcomes. Although new emerging therapies, such as transthyretin stabilizers (tafamidis) and gene-silencing agents (patisiran, vutrisiran), have been shown to improve survival by controlling disease progression, their efficacy markedly decreases at advanced stages [4].

The 2021 ESC recommendations for family screening in h-ATTR-CA [5] suggest the use of bone scintigraphy as part of the evaluation of asymptomatic ATTR mutation carriers (ATTR-MC). However, a European multicenter study [6] showed that conduction abnormalities can precede amyloid detection by bone scintigraphy in ATTR-MC, highlighting the limitations of first-line tests.

Given these limitations, CMR may play a pivotal role in their evaluation. It remains unclear whether myocardial signal alterations or cardiac functional changes at CMR in asymptomatic carriers of pathogenic TTR mutations precede overt clinical signs of systemic amyloidosis. The application of advanced CMR techniques, such as T1 mapping and myocardial strain analysis, appears promising in uncovering new biomarkers that might detect subclinical changes in the early stages of the disease [7,8]; in particular, ventricular and atrial myocardial strain analysis provides a quantitative approach to assessing myocardial deformation over the entire cardiac cycle.

The objective of our study is to characterize the morphofunctional changes and myocardial signal alterations detectable by CMR in ATTR-MC. These findings may distinguish them from normal individuals and potentially represent early signs of CA prior to the clinical manifestation of the disease.

## 2. Materials and Methods

### 2.1. Study Population

A retrospective analysis was conducted on a cohort of 40 subjects, divided into two distinct groups based on clinical presentation and diagnostic criteria: 20 patients with ATTR amyloidosis (Group 1—ATTR-CA) and 20 carriers of hereditary mutations in the TTR gene associated with amyloidosis (Group 2—ATTR-MC) without clinical signs, symptoms, or echocardiographic evidence of structural heart disease.

A control group of 20 age- and sex-matched patients with the ATTR-MC group, who underwent CMR for other indications with normal cardiac morphology and function and no myocardial signal abnormalities, was retrospectively enrolled (Group 3—Controls, C).

### 2.2. Inclusion Criteria

All patients in the ATTR-CA group presented left ventricular hypertrophy (LVH) at echocardiography, defined as a maximal wall thickness (MWT) > 13 mm. In cases where ATTR-CA was not histologically proven, the diagnosis was established using the non-invasive algorithm proposed by Gillmore et al. [9]. This approach requires the presence of heart failure with echocardiographic or CMR findings suggestive of amyloidosis, grade 2 or 3 cardiac uptake on technetium-99m PYP scintigraphy, and the absence of a monoclonal protein based on serum or urine immunofixation electrophoresis (IFE). Given that the Phe84Leu mutation is known to produce negative results on scintigraphy, alternative criteria were considered to assess potential cardiac involvement in these patients. Specifically, we relied on echocardiographic findings and conventional CMR parameters, including wall thickness, late gadolinium enhancement, and mapping values.

All ATTR-MC subjects, recruited among relatives of ATTR-CA patients, underwent genetic testing alongside echocardiography, myocardial scintigraphy, and serum/urine IFE analysis, which yielded negative results for amyloid deposition or abnormal protein expression.

**Figure 1:**
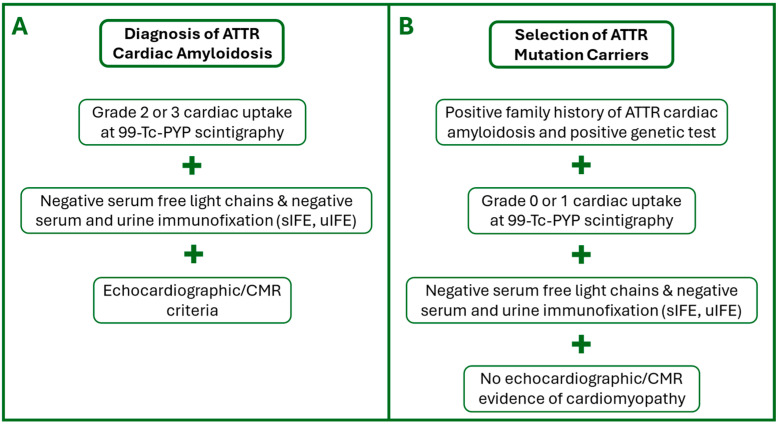
Diagnostic algorithm for patients with suspected ATTR-CA (**A**) and subjects with ATTR-MC (**B**). ATTR, Amyloid TransThyretin-Related; CA, Cardiac Amyloidosis; MC, Mutation Carriers; CMR, Cardiac Magnetic Resonance; sIFE, Serum Immunofixation Electrophoresis; uIFE, Urine Immunofixation Electrophoresis; 99mTc-PYP, Technetium PyroPhosphate.

The inclusion criteria are summarized in the flow charts shown in Figure 1.

### 2.3. Exclusion Criteria

Patients were excluded from this study if they met any of the following criteria: presence of cardiac devices (pacemakers or implantable cardioverter-defibrillators); poor image quality (e.g., inadequate breath-holding or significant artifacts); end-stage Chronic Kidney Disease, defined as an estimated glomerular filtration rate (eGFR) < 15 mL/min/1.73 m^2^; history of severe allergic reactions to contrast agents; pregnancy or lactation.

### 2.4. CMR Protocol

All CMRs were performed on a 3.0T scanner (Magnetom Vida, Siemens Medical System, Erlangen, Germany) using body and phased array coils.

The CMR protocol required the administration of a single dose of 0.15 mmol/Kg of Gadobutrol (Gadovist, Bayer Inc., Mississauga, ON, Canada) and included the following:

- T2-weighted Short Tau Inversion Recovery (T2-STIR) sequence acquired on the short axis (from base to apex, 10 slices at least) and on 2- and 4-chamber views (TR: 800; TE: 44 ms; TI: 220 ms; FA: 180°; Slice thickness: 6 mm; FoV: 360 mm; FoV Phase: 81.3%; Voxel size: 1.4 × 1.4 × 6 mm; Matrix: 135 × 256);
- Cine-Steady State Free Precession sequences for cineMR imaging, acquired on the short axis (from the base to the cardiac apex, 10 slices at least) and on 2-, 3-, and 4-chamber views (TR: 40.8 ms; TE: 1.49 ms; FA: 80°; Slice thickness: 6 mm; FoV: 360 mm; FoV Phase: 81.3%; Voxel size: 1.4 × 1.4 × 6.0 mm; Matrix: 191 × 256);
- Modified Look–Locker Inversion Recovery (MOLLI) sequence for nT1 mapping, acquired on three short-axis slices at basal, mid-ventricular, and apical views and one four-chamber view before (TR: 280.6 ms; TE: 1.12 ms; FA: 35°; Slice thickness: 8 mm; FoV: 360 mm; FoV Phase: 85.2%; Voxel size: 1.4 × 1.4 × 8 mm; Matrix 144 × 256) and 15 min after the administration of contrast media (TR: 360.6 ms; TE: 1.12 ms; FA: 35°; Slice thickness: 8 mm; FoV: 360 mm; FoV Phase: 85.2%; Voxel size: 1.4 × 1.4 × 8 mm; Matrix 144 × 256);
- Contrast-enhanced Inversion Recovery T1-weighted images were acquired from 10 to 15 min after contrast media administration, during breath-hold at end-diastole in the short axis (from the base to the cardiac apex, 10 slices at least), and on 2-, 3-, and 4-chamber views (TR: 750 ms; TE: 2.01 ms; TI: 300–350 ms; FA: 20°; Slice thickness: 6 mm; FoV: 360 mm; FoV Phase: 85.2%; Voxel size: 1.4 × 1.4 × 8 mm; Matrix 154 × 256) for LGE imaging.

Field inhomogeneity correction, noise reduction, and motion correction were performed using the algorithms incorporated in the package with standard settings.

### 2.5. Image Analysis

The image analysis was conducted by two radiologists (L.C., 5 years of experience, and D.F., 7 years of experience) in consensus, utilizing a dedicated postprocessing software (Cvi42 v6.0.2, Circle Cardiovascular Imaging, Calgary, AB, Canada). Atrial and ventricular volumes, along with derived parameters, were measured using cineMR images.

Atrial and ventricular volumetric measurements, along with derived indices, were obtained from cineMR images. The endocardial and epicardial borders of the left ventricle (LV) were delineated semi-automatically on both short- and long-axis views, and all volumetric parameters were indexed to body surface area (BSA).

LV strain analysis was conducted through CMR feature tracking based on cineMR images in multiple planes. The resulting myocardial contours were reviewed visually, adjusted to correct tracking inaccuracies, and reanalyzed as necessary, following previously established protocols. Global circumferential strain (GCS) and global longitudinal strain (GLS) were calculated as the average of three repeated measurements.

Left atrial (LA) strain evaluation was carried out using the same feature-tracking methodology. Endocardial LA borders were outlined in horizontal and vertical long-axis cineMR views at the frame following atrial contraction and automatically propagated across the cardiac cycle. All generated contours underwent visual inspection, correction if required, and final validation by the operators. LA reservoir, conduit, and booster pump functions were quantified through longitudinal strain analysis corresponding to the reservoir (LA RS), conduit (LA CS), and booster pump (LA BPS) phases, respectively. Each analysis was repeated three times, and mean values were reported.

For T1 mapping, contours were drawn around the endocardial and epicardial borders on pre-contrast and 15-min post-contrast images. A region of interest (ROI) was positioned within the LV cavity to extract blood pool T1 values, and a reference marker was placed at the anterior interventricular junction. Native T1 (nT1) values and ECV were automatically generated by the software for each segment according to the AHA model [10], according to the formula described elsewhere [11]. Hematocrit levels were collected within 24 h prior to imaging.

For reproducibility purposes, the identification of strain characteristics in all CMR studies was repeated by two blinded investigators; inter-observer and intra-observer variability of LV and LA strain was assessed for all the subjects in the study population.

**Figure 2:**
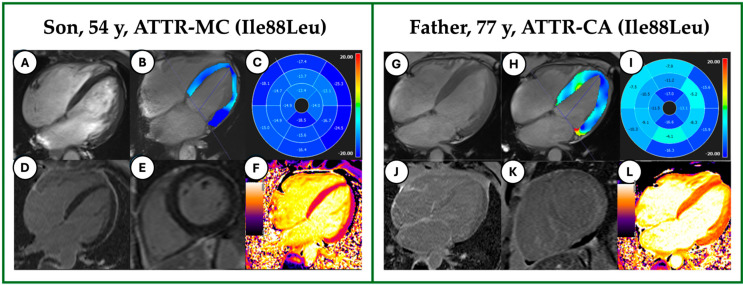
Case Study: Comparison among Relatives. On the left are the CMR images of the son, 54 y, carrier of the mutation Ile88Leu of the *TTR* gene, with a preserved EF (55%) and a normal MWT (10 mm) in the cine images (**A**), a mildly impaired GLS in the strain analysis (−16.1 ± 4.3%, (**B**,**C**)), no LGE (**D**,**E**) and normal nT1 values (1235 ± 40; normal range: 1153–1307 ms, (**F**)). On the right are the CMR images of the Father, 77 y, with CA related to the same mutation, with a reduced EF (35%), a moderate LV hypertrophy (18 mm) in the cine images (**G**), a severely impaired GLS in the strain analysis (−9 ± 5.9%, (**H**,**I**)), typical LGE pattern (**J**,**K**) and incremented nT1 values (1435 ± 30, (**L**)). ATTR, Amyloid TransThyretin Related; MC, Mutation Carrier; Ile, Ileleucin; Leu, Leucine; CA, Cardiac Amyloidosis; CMR, Cardiac Magnetic Resonance; TTR, TransThyretin; EF, Ejection Fraction; MWT, Maximum Wall Thickness; LGE, Late Gadolinium Enhancement; nT1, Native T1.

Examples of image analysis are depicted in the case study presented in Figure 2.

### 2.6. Statistical Analysis

Categorical variables were summarized as frequencies and percentages, while continuous data were expressed as means with standard deviations. The assumption of normality was assessed using both the Kolmogorov–Smirnov and Shapiro–Wilk tests. For variables not following a normal distribution, data were presented as medians with interquartile ranges (IQR). Group comparisons for non-normally distributed data were carried out using the Mann–Whitney U test or the Kruskal–Wallis test, as appropriate.

For continuous variables exhibiting normal distribution, independent sample *t*-tests were used to compare means between the two groups. When comparing more than two groups, one-way analysis of variance (ANOVA) was applied, followed by Bonferroni-adjusted post hoc tests for pairwise comparisons.

Associations between parameters were assessed using Pearson’s correlation coefficient for normally distributed data and Spearman’s rank correlation for non-normal variables. The strength of correlations was interpreted as follows: negligible (0), slight (0.01–0.20), fair (0.21–0.40), moderate (0.41–0.60), good (0.61–0.80), and excellent (0.81–1.00).

Reproducibility of strain measurements was assessed through intraclass correlation coefficients (ICC), with values between 0.40 and 0.75 indicating fair to good reliability, and values above 0.75 are considered excellent.

Receiver operating characteristic (ROC) curve analysis was performed to evaluate the diagnostic performance of atrial and ventricular strain parameters in distinguishing between ATTR-MC and healthy controls. Optimal threshold values were identified using Youden’s index.

All statistical analyses were conducted using SPSS software (version 30.0, IBM Corp., Armonk, NY, USA). A *p*-value less than 0.05 was considered statistically significant.

## 3. Results

### 3.1. Demographic and Clinical Characteristics

No significant differences were observed between ATTR-MC and controls in terms of age, body surface area (BSA), heart rate (HR), or blood pressure (systolic and diastolic).

Patients in the ATTR-CA group were significantly older (77.2 ± 8.8 years) compared to ATTR-MC (52.7 ± 10.3 years; *p* < 0.05) and controls (47.0 ± 12.2 years; *p* < 0.05). All patients in the ATTR-CA group had positive technetium-99m-PYP scintigraphy, with a mean visual score of 2.74 ± 0.44, confirming the diagnosis of CA.

**Figure 3:**
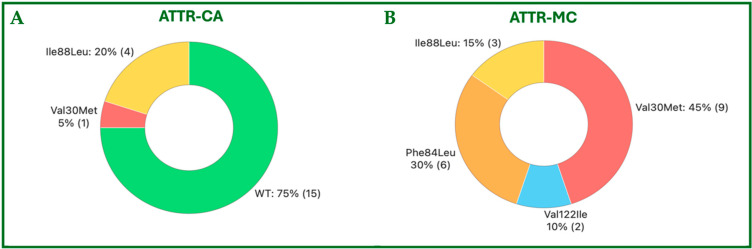
Graphical representation of the distribution of ATTR mutations in the study population: (**A**) in patients with ATTR-CA and (**B**) in subjects with ATTR-MC. ATTR, Amyloid TransThyretin Related; CA, Cardiac Amyloidosis; MC, Mutation Carriers; ILE, Ileleucin; Leu, Leucine; Phe, phenylalanine; Val, Valine; Met, Methionine.

The demographic and clinical characteristics are summarized in Table 1, whereas the TTR mutations identified in the study population are reported in Figure 3.

Additional information regarding medical therapies and laboratory tests related to early signs of heart failure, such as troponin and NT-proBNP, is provided in the Supplementary Materials, in Table S1.

### 3.2. CMR Features

In the ATTR-MC group, no pathological phenotype was detected in the standard analysis of CineMR images, without a significant alteration of LV volume and ejection fraction (EF) compared to controls (indexed LV end-diastolic volume—LV EDVi: 74.7 ± 12.8 vs. 74.0 ± 11.5 mL/m^2^, *p* = 1.000, LV EF: 59.2 ± 5.9% vs. 61.3 ± 3.8%, *p* = 0.703), MWT, and in myocardial mass index (MMi).

The ATTR-CA group demonstrated significant increases in LV EDVi (85.9 ± 26.0 mL/m^2^), along with a marked reduction in LV EF (43.4 ± 12.1%), compared to ATTR-MC and controls (*p*-value < 0.05). The ATTR-CA group also showed substantial increases in MWT (19.7 ± 3.9 mm) and MMi, indicating severe hypertrophy.

The CMR features are reported in Table 2.

### 3.3. CMR Tissue Characterization

In the ATTR-MC group, no significant differences were identified in nT1 (1234.8 ± 38.1 ms vs. 1238.3 ± 45.3 ms, *p* = 1.000) or ECV (28.7 ± 3.9% vs. 29.1 ± 3.1%, *p* = 1.000) values compared to controls, neither subjects with values outside the normal range were identified.

There were no individuals with areas of myocardial edema or LGE in both ATTR-MC and controls.

ATTR-CA patients exhibited markedly elevated nT1 (1364.3 ± 66.5 ms) and ECV (49.2 ± 12.6%) compared to ATTR-MC and controls, consisting of the interstitial expansion due to amyloid accumulation in myocardial tissue.

**Figure 4:**
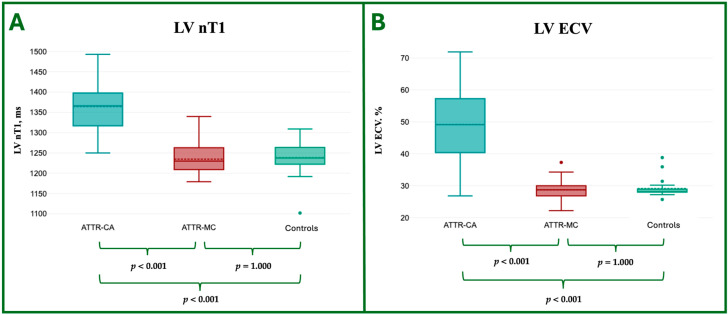
Box plot representation of the ANOVA comparison of nT1 (**A**) and ECV (**B**) among ATTR-CA, ATTR-MC, and Controls groups. ATTR, Amyloid TransThyretin Related; CA, Cardiac Amyloidosis; MC, Mutation Carriers; nT1, Native; ECV, ExtraCellular Volume.

The T1 mapping and ECV value comparison are reported in Table 3 and in Figure 4.

### 3.4. CMR LV and LA Strain Analysis

Despite preserved LV volumes and ejection fraction, ATTR-MC exhibits a significant reduction in LV GLS (−15.6 ± 2.1%) compared to HC (−18.4 ± 1.0%; *p* < 0.001). GCS was also slightly reduced but not statistically different from HC (−17.1 ± 2.6% vs. −18.6 ± 2.2%, *p* = 0.345).

**Figure 5:**
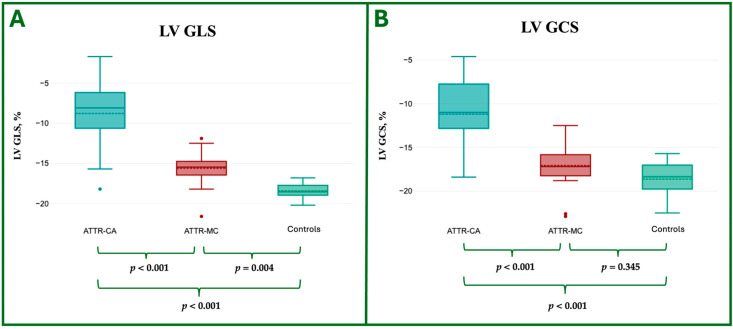
Box plot representation of the ANOVA comparison of LV strain parameters—specifically GLS (**A**) and GCS (**B**)—among ATTR-CA, ATTR-MC, and HC groups. LV, Left Ventricle; ATTR, Amyloid TransThyretin Related; CA, Cardiac Amyloidosis; MC, Mutation Carriers; C, Controls; GLS, Global Longitudinal Strain; GCS, Global Circumferential Strain.

**Figure 6:**
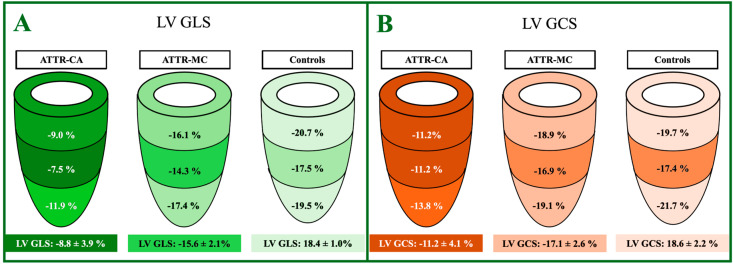
Graphical representation of the ANOVA comparison of LV strain parameters—specifically GLS (**A**) and GCS (**B**)—among ATTR-CA, ATTR-MC, and HC groups. LV, Left Ventricle; ATTR, Amyloid TransThyretin Related; CA, Cardiac Amyloidosis; MC, Mutation Carriers; C, Controls; GLS, Global Longitudinal Strain; GCS, Global Circumferential Strain.

The CMR LV strain analysis features are reported in Table 4 and shown in Figure 5 and Figure 6.

Similarly, despite preserved LA atrial volumes (systolic and diastolic), ATTR-MC demonstrated impaired LA strain values compared to controls. There was a significant reduction in LA RS (−19.8 ± 2.8% vs. −22.8 ± 3.5%, *p* = 0.007) and LA CS (−9.4 ± 1.9% vs. −14.8 ± 3.2%, *p* < 0.001) with a significant increase in LA BPS (10.5 ± 2.2% vs. 8.0 ± 3.0%, *p* = 0.005).

**Figure 7:**
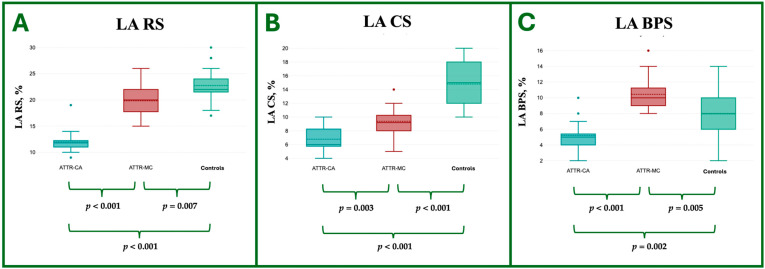
Box plot representation of the ANOVA comparison of LA strain parameters—specifically RS (**A**), CS (**B**), and BPS (**C**)—among ATTR-CA, ATTR-MC, and HC groups. LA, Left Atrial; ATTR, Amyloid TransThyretin Related; CA, Cardiac Amyloidosis; MC, Mutation Carriers; C, Controls; RS, Reservoir Strain; CS, Conduit Strain; BPS, Booster Pump Strain.

The CMR LA strain analysis features are reported in Table 4 and shown in Figure 7.

**Figure 8:**
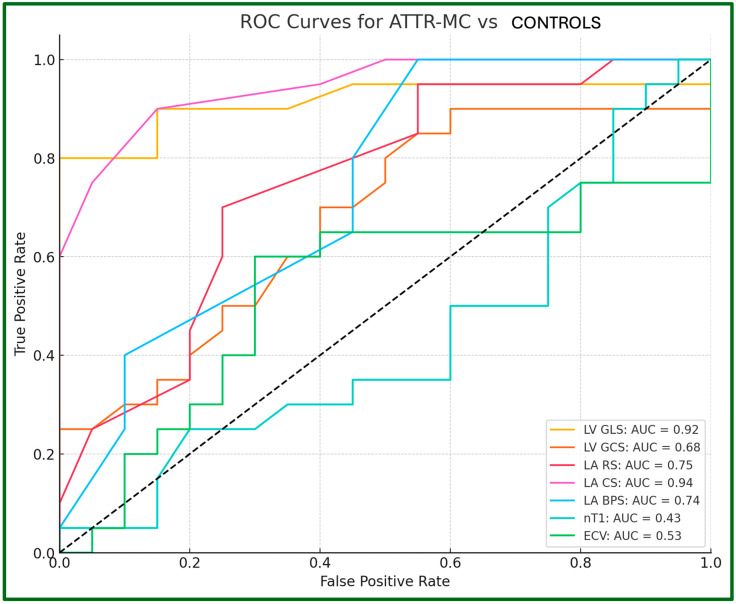
ROC Curves for Diagnostic Accuracy of LV/LA strain parameters and nT1/ECV values between ATTR-MC and Controls. Each curve is annotated with the corresponding AUC value, showcasing the performance of each parameter. Optimal thresholds for sensitivity and specificity were determined using Youden’s index. ROC, Receiver Operating Characteristic; ATTR, Amyloid TransThiretin related; MC, Mutation Carriers; HC, Healthy Controls; LV, Left Ventricle; LA, Left Atrium; GLS, Global Longitudinal Strain; GCS, Global Circumferential Strain; RS, Reservoir Strain; CS, Conduit Strain; BPS, Booster Pump Strain; nT1, native T1; ECV, ExtraCellular Volume; AUC, Area Under the Curve.

We assessed the capability of LV and LA strain parameters, nT1 and ECV values, to distinguish between ATTR-MC and controls using receiver operating characteristic (ROC) curves analysis, evaluating the area under the curve (AUC), as reported in Figure 8. LA CS and LV GLS demonstrated excellent accuracy with an AUC of 0.94 and 0.92, respectively.

LA BPS and LA exhibited fair accuracy with an AUC of 0.74 and 0.75, respectively. LA GCS was the only parameter with poor accuracy, with an AUC of 0.68. nT1 and ECV failed to distinguish the two groups with an AUC of 0.43 and 0.53, respectively.

## 4. Discussion

Our study demonstrated that, although CA is associated with an increase in myocardial nT1 and ECV values, ATTR-MC does not exhibit alterations in myocardial relaxometric properties. However, despite preserved LV myocardial wall thickness, volumes, ejection fraction, and LA volumes, ATTR-MC showed reduced LV GLS, LA RS, and LA CS, as well as increased LA BPS compared to HC. Moreover, those contractility alterations in ATTR-MC during strain analysis mirror those seen in CA patients, albeit with milder severity.

### 4.1. Tissue Characterization

In recent years, T1 mapping has emerged as the reference non-invasive tool for diagnosing CA in cases of myocardial hypertrophy of uncertain origin [12]. Indeed, nT1 and ECV values elevation occurs in CA due to interstitial space expansion associated with misfolded TTR protein accumulation and may also be observed in patients with systemic amyloidosis without ventricular hypertrophy or LGE [13].

ECV values in CA can reach approximately 54%, compared to normal levels of 20–26% and mildly elevated values of 27–31% in diffuse fibrotic conditions such as aortic stenosis and hypertrophic cardiomyopathy (HCM) [14]. T1 mapping has shown superior diagnostic accuracy compared to LGE in detecting CA, as nT1 values serve as a surrogate marker of both fibrosis and amyloid deposition [15].

However, the role of T1 mapping in the early diagnosis of ATTR-CA patients without myocardial hypertrophy remains poorly investigated and is still to be clarified.

In their studies on ATTR amyloidosis, Martinez-Naharro et al. [16] and Fontana et al. [17] investigated nT1/ECV differences among wt-ATTR, h-ATTR, and HCM patients. Their cohorts included 8 and 12 ATTR-MC patients, respectively, without evident cardiac involvement on echocardiography or myocardial scintigraphy. In these subgroups, no pathological alterations in nT1 or ECV values were found in ATTR-MC patients.

Our study confirmed that both nT1 (1234.8 ± 38.1 ms and 1238.3 ± 45.3 ms) and ECV values (28.7 ± 3.9% and 29.1 ± 3.1%) did not differ between ATTR-MC and controls (*p* = 1.000 for both parameters), suggesting the absence of detectable extracellular TTR deposition by CMR. This could be explained by the fact that, in ATTR-MC, any potential amyloid deposition in the extracellular space remains insufficient to induce significant fluid accumulation, a key factor contributing to increased nT1 and ECV values.

In the study population, no ATTR-MC exhibited myocardial edema or LGE, excluding the possibility of cardiac inflammation or fibrosis, whereas all ATTR-CA patients showed LV LGE areas.

These findings support the need for additional CMR-derived features to improve early diagnosis and characterization of cardiac involvement in patients at risk of developing ATTR-CA.

### 4.2. Ventricular Strain

LV strain analysis has emerged as a key tool for detecting subclinical myocardial dysfunction. By quantifying myocardial deformation, it identifies subtle impairments that may precede functional decline, aiding in the early diagnosis of cardiomyopathies [18].

In our cohort, ATTR-MC patients showed impaired LV GLS despite the absence of hypertrophy and normal nT1/ECV values. This is notable, as CA-related contractile dysfunction is primarily driven by amyloid-induced stiffness, leading to a restrictive pattern [1]. In ATTR-MC, early systolic dysfunction may reflect additional pathogenic mechanisms affecting cardiomyocytes, potentially preceding impairment due to interstitial amyloid infiltration.

In ATTR-MC, the early systolic dysfunction could reflect an additional pathogenetic mechanism that could precede the interstitial space expansion due to amyloid fibril deposition. In their comprehensive review of CA.

Ruberg FL et Berk JL [19] suggested that neuropathy or conduction disturbances caused by the toxic effects of pre-fibrillar transthyretin aggregates may occur before significant amyloid deposition. Histological evidence from in vivo nerve biopsies of asymptomatic Val30Met carriers points to pre-fibrillar TTR aggregates as key drivers of early nerve injury, even before clinical symptoms appear. In these carriers, nonfibrillar TTR aggregates triggered inflammatory responses and signs of cell stress.

In ATTR-MC, GCS was not significantly reduced compared to HC, likely because GLS is the first to decline with subendocardial damage, whereas GCS is influenced by subepicardial or transmural injury [5,20,21].

Selective GLS reduction suggests predominant subendocardial impairment in the early stages of CA, likely due to its vulnerability to amyloid deposition, lower capillary density, and higher ventricular filling pressures. GLS also detects early myocardial dysfunction missed by EF measurements, as echocardiography and CMR showed preserved EF [22].

These findings align with studies on LV strain impairment in other cardiomyopathy mutation carriers, even without significant echocardiographic or CMR abnormalities [23,24,25]. Vijapurapu et al. reported LV GLS impairment in Fabry disease without nT1 reduction, suggesting that mechanical dysfunction preceded evidence of sphingolipid deposition on the T1 map [26].

Furthermore, in our study, the optimal LV GLS cut-off value to distinguish ATTR-MC from HC was −16.6%, with an AUC = 0.92, indicating excellent performance. We also confirmed LV strain impairment in ATTR-CA, consistent with previous studies [27]. The Youden test identified −13.1% as the optimal LV GLS cut-off to differentiate ATTR-CA from ATTR-MC and HC (AUC = 0.94), also showing high discriminatory power. A relative apical sparing pattern has been confirmed, characterized by a basal-apical gradient (“cherry-on-top” pattern).

These findings highlight the potential additional role of strain analysis in this clinical setting, complementing traditional CMR features, such as LGE, ventricular volumes, and mapping values.

### 4.3. Atrial Strain

LA strain enables phasic analysis of LA function, divided into reservoir, conduit, and booster pump phases, accounting for approximately 50%, 30%, and 20% of LV filling in healthy subjects, respectively [28]. LA strain is altered in all cardiac diseases with diastolic dysfunction, often preceding systolic impairment [29].

ATTR-CA exhibits more advanced LA structural and functional remodeling than other non-ischemic diseases [30], suggesting LA strain features as a potential tool for early diagnosis and severity classification. In our cohort of ATTR-MC subjects, LA RS and LA CS were significantly reduced, while LA BPS increased, indicating a compensatory mechanism at the early stage of cardiac involvement to maintain ventricular filling despite subclinical diastolic dysfunction.

The Youden test identified an optimal LA CS cut-off of −11.0% (AUC = 0.94) to distinguish between ATTR-MC and HC, whereas LA RS and LA BPS showed lower discriminatory performance (AUC = 0.75 and 0.74, respectively).

LA strain is significantly affected in CA due to LV diastolic dysfunction and amyloid infiltration in the atrial wall [31]. In ATTR-MC, these alterations may reflect a combination of early extracellular TTR deposition, undetectable by LGE or mapping, and compensatory responses to ventricular dysfunction.

These findings align with previous studies on CA and other storage diseases, such as Fabry disease. Meucci et al. found LA strain independently correlated with ATTR, unlike LV GLS [32]. Halfmann et al. showed that the LA strain effectively differentiated early Fabry disease from HC, even when volumetric parameters and T1 mapping were inconclusive [33].

These findings highlight the potential additional role of strain analysis, both atrial and ventricular, in this clinical setting, complementing traditional CMR features such as LGE, ventricular volumes, and mapping values. Notably, the use of CMR-based strain analysis in ATTR-MC has been scarcely investigated, especially in the absence of overt cardiac involvement. Our study contributes novel evidence that even in the early, preclinical phase of the disease, LV strain, particularly GLS, and LA strain, particularly LA CS, may detect subtle myocardial dysfunction not captured by conventional imaging markers. This underscores the value of integrating strain analysis into the CMR assessment of mutation carriers, enhancing early diagnostic sensitivity and potentially guiding closer monitoring and timely therapeutic interventions.

While both LA and LV strain parameters were impaired in our cohort, a direct comparative analysis between the degree of LA and LV strain reduction was not performed. Future studies are warranted to explore whether LA dysfunction precedes or exceeds that of the LV, as this may provide further insights into the temporal and functional relationship between atrial and ventricular involvement in this clinical setting.

### 4.4. Study Limitations

One of the key limitations of this study is the relatively small sample size, which may constrain the generalizability of the findings and reduce the power to detect more subtle or nuanced differences between groups. A larger cohort could enhance the robustness of the conclusions and potentially reveal additional insights into the studied parameters. Observer variability represents another potential limitation.

Indeed, while reproducibility was assessed, the LA strain measurements demonstrated slightly lower ICC values compared to the LV strain. This finding suggests that further standardization and protocol refinement may be necessary to improve measurement consistency and reduce variability across different operators or imaging centers.

Another limitation is the retrospective design of this study. This approach was chosen due to the rarity of the condition and the availability of pre-existing, clinically acquired imaging and biomarker data.

Lastly, the lack of longitudinal follow-up in the study design precludes the evaluation of the prognostic significance of the detected early strain abnormalities. A follow-up investigation would be essential to determine whether these early subclinical findings are predictive of the eventual onset of overt cardiac amyloidosis in individuals carrying pathogenic mutations. Such an approach could help elucidate the temporal relationship between strain alterations and disease progression, contributing to improved risk stratification and clinical management.

## 5. Conclusions

A reduction in ventricular systolic strain, together with an increase in LA BPS, allowed for the identification of subclinical cardiac contractility disorders among ATTR-MC patients compared to controls. CMR-derived strain analysis, therefore, represents a powerful tool for identifying myocardial dysfunction in ATTR mutation carriers, with the potential to significantly enhance clinical management.

Longitudinal studies will be essential to evaluate the prognostic impact of these alterations and guide personalized therapeutic decisions.

## Figures and Tables

**Table 1:** Demographic and Clinical Variables.

	Group 1 ATTR-CA	Group 2 ATTR-MC	Group 3 Controls	CA vs. MC *p*-Value	CA vs. C *p*-Value	MC vs. C *p*-Value
Age, y	77.2 ± 8.8	52.7 ± 10.3	47.0 ± 12.2	<0.001 **	<0.001 **	0.274
Male, n (%)	11 (55%)	8 (40%)	6 (30%)	0.527	0.200	0.741
BSA, m^2^	1.79 ± 0.19	1.82 ± 0.19	1.70 ± 0.17	1.000	0.334	0.107
HR, bpm	76.2 ± 11.0	76.4 ± 10.3	80.0 ± 10.7	1.000	0.521	0.573
sBP, mmHg	120.9 ± 12.0	120.9 ± 10.5	120.0 ± 11.2	1.000	1.000	1.000
dBP, mmHg	73.3 ± 6.3	73.3 ± 6.5	80.0 ± 6.4	1.000	1.000	1.000
eGFR, mL/min	61.6 ± 16.8	88.9 ± 16.6	100.0 ± 16.7	<0.001 **	<0.001 **	0.036 *
Positivity to [99mTc] PYP scintigraphy, n (%)	20 (100%)	0 (0%)	N/A	<0.001 **	N/A	N/A
Scintigraphy visual score, n	2.74 ± 0.44	0.0 ± 0	N/A	<0.001 **	N/A	N/A
Positivity to Urine/Serum IFE, n (%)	0 (0%)	0 (0%)	N/A	1.000	N/A	N/A

Values are mean ± SD or n (%) unless otherwise indicated. * *p*-value < 0.05 and ** *p*-value < 0.01. ATTR, Amyloid TransThyretin Related; CA, Cardiac Amyloidosis; MC, Mutation Carriers; C, Controls; BSA, Body Surface Area; HR, Heart Rate; sBP, systolic blood pressure; dBP, diastolic blood pressure; eGFR, estimated Glomerular Filtration Rate; 99mTC, Technetium-99m; PYP, Pyrophosphate; IFE, Immunofixation Exam.

**Table 2:** CMR Features.

	Group 1 ATTR-CA	Group 2 ATTR-MC	Group 3 HC	CA vs. MC *p*-Value	CA vs. HC *p*-Value	MC vs. HC *p*-Value
LV EDVi, mL/m^2^	85.9 ± 26.0	74.7± 12.8	74.0 ± 11.5	0.165	0.124	1
LV ESVi, mL/m^2^	50.4 ± 22.4	30.7 ± 7.6	28.8 ± 5.8	<0.001 **	<0.001 **	1
LV EF, %	43.4 ± 12.1	59.2 ± 5.9	61.3 ± 3.8	<0.001 **	<0.001 **	0.703
LV MWT, mm	19.7 ± 3.9	9.9 ± 1.62	9.5 ± 1.6	<0.001 **	<0.001 **	1
LV MMi, g/m^2^	86.9 ± 28.3	46.5 ± 9.4	43.2 ± 8.5	<0.001 **	<0.001 **	1
LA EDVi, mL/m^2^	51.1 ± 19.6	14.3 ± 6.5	11.7 ± 3.4	<0.001 **	<0.001 **	1
LA ESVi, mL/m^2^	65.6 ± 25.5	32.0 ± 12.0	32.1 ± 9.6	<0.001 **	<0.001 **	1

Values are mean ± SD or n (%) unless otherwise indicated. ** *p*-value < 0.01. ATTR, Amyloid Transthyretin Related; CA, Cardiac Amyloidosis; MC, Mutation Carriers; HC, Healthy Controls; LV, Left Ventricle; EDVi, Indexed End-Diastolic Volume; ESVi, Indexed End Systolic Volume; EF, Ejection Fraction; LA, Left Atrium.

**Table 3:** CMR Tissue Characterization.

	Group 1 ATTR-CA	Group 2 ATTR-MC	Group 3 HC	CA vs. MC *p*-Value	CA vs. HC *p*-Value	MC vs. HC *p*-Value
LV Edema, n (%)	0/20 (0%)	0/20 (0%)	0/20 (0%)	N/A	N/A	N/A
LV LGE, n (%)	20/20 (100%)	0/20 (0%)	0/20 (0%)	N/A	N/A	N/A
LV nT1, ms	1364.3 ± 66.5	1234.8 ± 38.1	1238.3 ± 45.3	<0.001 **	<0.001 **	1
LV T2, ms	43.5 ± 2.8	42.7 ± 5.1	41.1 ± 3.5	1	0.193	0.608
LV ECV, %	49.2 ± 12.6	28.7 ± 3.9	29.1 ± 3.1	<0.001 **	<0.001 **	1

Values are mean ± SD or n (%) unless otherwise indicated. ** *p*-value < 0.01. Abbreviations: ATTR, Amyloid Transthyretin Related; CA, Cardiac Amyloidosis; MC, Mutation Carriers; C, Controls; LV, Left Ventricle; LGE, Late Gadolinium Enhancement; nT1, Native T1; ECV, ExtraCellular Volume.

**Table 4:** CMR Strain analysis.

	Group 1 ATTR-CA	Group 2 ATTR-MC	Group 3 Controls	CA vs. MC *p*-Value	CA vs. HC *p*-Value	MC vs. HC *p*-Value
LV GLS, %	−8.8 ± 3.9	−15.6 ± 2.1	−18.4 ± 1.0	<0.001 **	<0.001 **	0.004 **
LV BAS-LS, %	−9.0 ± 5.8	−16.1 ± 4.3	−20.7 ± 1.7	<0.001 **	<0.001 **	0.003 **
LV MID-LS, %	−7.5 ± 5.5	−14.3 ± 3.9	−17.5 ± 2.0	<0.001 **	<0.001 **	0.046 *
LV API-LS, %	−11.9 ± 5.8	−17.4 ± 4.0	−19.5 ± 2.9	<0.001 **	<0.001 **	0.39
LV GCS, %	−11.2 ± 4.1	−17.1 ± 2.6	−18.6 ± 2.2	<0.001 **	<0.001 **	0.345
LV BAS-CS, %	−11.2 ± 3.1	−18.9 ± 2.7	−19.7 ± 3.1	<0.001 **	<0.001 **	1
LV MID-CS, %	−11.2 ± 4.6	−16.9 ± 2.7	−17.4 ± 2.3	<0.001 **	<0.001 **	1
LV API-CS, %	−13.8 ± 5.6	−19.1 ± 3.6	−21.7 ± 3.2	<0.001 **	<0.001 **	0.189
LA RS, %	−12.0 ± 2.2	−19.8 ± 2.8	−22.8 ± 3.5	<0.001 **	<0.001 **	0.007 **
LA CS, %	−6.8 ± 1.8	−9.4 ± 1.9	−14.8 ± 3.2	0.003 **	<0.001 **	<0.001 **
LA BPS, %	5.2 ± 1.7	10.5 ± 2.2	−8.0 ± 3.0	<0.001 **	0.002 **	0.005 **

Values are mean ± SD or n (%) unless otherwise indicated. * *p*-value < 0.05 and ** *p*-value < 0.01. ATTR, Amyloid TransThyretin; CA, Cardiac Amyloidosis; MC, Mutation Carriers; C, Controls; LV, Left Ventricle; GLS, Global Longitudinal Strain; BAS, Basal; API, Apical; GCS, Global Circumferential Strain; LA, Left Atrial; RS, Reservoir Strain; CS, Conduit Strain; BPS, Booster Pump Strain.

## Data Availability

The data supporting the findings of this study are not publicly available due to privacy and ethical restrictions.

## Supplementary Materials

The following supporting information can be downloaded at: https://www.mdpi.com/article/10.3390/jcdd12050172/s1, Table S1: Supplementary Clinical Variables—Laboratory Tests and Medications.

## Abbreviations

The following abbreviations are used in this manuscript:

AUC	Area Under the Curve;
ATTR	Amyloid TransThyretin Related;
BPS	Booster Pump Strain;
C	Controls;
CA	Cardiac Amyloidosis;
CMR	Cardiac Magnetic Resonance;
CS	Conduit Strain;
ECG	Electrocardiogram;
ECV	Extracellular Volume;
EF	Ejection Fraction;
eGFR	Estimated Glomerular Filtration Rate;
GCS	Global Circumferential Strain;
GLS	Global Longitudinal Strain;
h-ATTR	Hereditary ATTR;
ICC	Intraclass Correlation Coefficient;
IFE	Immunofixation Electrophoresis;
LA	Left Atrium;
LGE	Late Gadolinium Enhancement;
LV	Left Ventricle;
LVH	Left Ventricular Hypertrophy;
MC	Mutation Carriers;
MMi	Myocardial Mass Index;
MOLLI	Modified Look–Locker Inversion Recovery;
MWT	Maximal Wall Thickness;
PYP	Pyrophosphate;
ROC	Receiver Operator Characteristics;
RS	Reservoir Strain;
TTR	Transthyretin;
Wt-ATTR	Wild-Type ATTR.
