# Supplementary material for: Subclinical Cardiac Involvement in Asymptomatic ATTR Mutation Carriers: Insights from Cardiac MRI, Myocardial Strain, and Mapping Techniques
Source: J Cardiovasc Dev Dis. 2025 May 1;12(5):172. doi: 10.3390/jcdd12050172 (PMC12112504; doi:10.3390/jcdd12050172)
Supplement: Supplementary file 1 [file jcdd-12-00172-s001.zip › jcdd-3554002-supplementary.pdf]

Table S1. Supplementary Clinical Variables – Laboratory Tests and Medications

	Group 1 ATTR-CA	Group 2 ATTR-MC	Group 3 Controls
**Troponin I, ng/l***	62.8 ± 30.0	5.2 ± 2.9	N/A
**NT-pro-BNP, pg/ml***	3074 ± 2226	57.3 ± 54	N/A
**Anti-amyloid drugs, n (%)**	10/20 (50%)	3/20 (15%)	0/20 (0%)
**Antihypertensives, n (%)**	7/20 (35%)	2/20 (10%)	0/20 (0%)
**Antiarrhythmics, n (%)**	1/20 (5%)	0/20 (0%)	0/20 (0%)
**Antiplatelets, n (%)**	1/20 (5%)	0/20 (0%)	0/20 (0%)
**Anticoagulants, n (%)**	3/20 (15%)	0/20 (0%)	0/20 (0%)
**Hypoglycemic agents, n (%)**	3/20 (15%)	0/20 (0%)	0/20 (0%)
**Hypolipidemic agents, n (%)**	5/20 (25%)	0/20 (0%)	0/20 (0%)

Values are mean ± SD or n (%), unless otherwise indicated.

ATTR Amyloid TransThyretin Related; CA Cardiac Amyloidosis; MC Mutation Carriers; NT-pro-BNP N-terminal pro–B-type Natriuretic Peptide.

*Normal values for our Center: Troponin I < 34 ng/l; NT-pro-BNP < 125 pg/ml.
